# Catechin synergistically potentiates mast cell-stabilizing property of caffeine

**DOI:** 10.1186/s13223-020-00502-5

**Published:** 2021-01-06

**Authors:** Misaki Yashima, Yukine Sato, Itsuro Kazama

**Affiliations:** Miyagi University, School of Nursing, 1-1 Gakuen, Taiwa-cho, Kurokawa-gun, Miyagi 981-3298 Japan

**Keywords:** Caffeine, Catechin, Exocytosis, Mast cells, Mast cell, Stabilizing property

## Abstract

Caffeine and catechin, contained in coffee and tea, are commonly consumed substances worldwide. Studies revealed their health promoting functions, such as anti-oxidant, anti-cancer and anti-bacterial properties. Additionally, studies also revealed their roles in ameliorating the symptoms of allergic disorders, indicating their anti-allergic properties. In the present study, using the differential-interference contrast (DIC) microscopy, we examined the effects of caffeine and catechin on the degranulation from rat peritoneal mast cells. Both caffeine and catechin dose-dependently decreased the numbers of degranulating mast cells. At concentrations equal to or higher than 25 mM, caffeine and catechin markedly suppressed the numbers of degranulating mast cells. In contrast, at relatively lower concentrations, both substances did not significantly affect the numbers of degranulating mast cells. However, surprisingly enough, low concentrations of catechin (1, 2.5 mM) synergistically enhanced the suppressive effect of 10 mM caffeine on mast cell degranulation. These results provided direct evidence for the first time that caffeine and catechin dose-dependently inhibited the process of exocytosis. At relatively lower concentrations, caffeine or catechin alone did not stabilize mast cells. However, low concentrations of catechin synergistically potentiated the mast cell-stabilizing property of caffeine.

**To the editor,**

Caffeine, a psychoactive alkaloid contained in coffee, and catechin, a polyphenolic flavonoid in tea, are commonly consumed substances worldwide [1, 2]. Previous studies revealed their health promoting functions, including anti-oxidant, anti-cancer and anti-bacterial properties [3, 4]. Additionally, both in humans or experimental animal models, recent studies also revealed their roles in ameliorating the symptoms of allergic disorders, such as bronchial asthma and anaphylaxis [5–8]. These studies indicated anti-allergic properties of caffeine and catechin, showing their additional pharmacological potency. In our previous studies, by continuously monitoring the process of exocytosis in mast cells, we provided in vitro evidence that anti-allergic drugs, anti-microbial drugs and corticosteroids exert mast cell-stabilizing properties [9–13]. In the present study, to elucidate the mechanisms underlying the anti-allergic properties of caffeine and catechin, we directly examined their effects on the degranulation from rat peritoneal mast cells.

In mast cells isolated from the peritoneal cavity of male Wistar rats (CLEA Japan Inc., Tokyo, Japan), we externally induced exocytosis by compound 48/80 (Sigma-Aldrich; final concentration 10 μg/ml) in the presence or absence of caffeine or catechin. Caffeine anhydrous, purchased from Wako Pure Chem Ind. (Osaka, Japan), was dissolved in the external solution at the final concentrations of 1 mM (194 μg/ml), 5 mM (971 μg/ml), 10 mM (1.94 mg/ml), 25 mM (4.86 mg/ml), 50 mM (9.71 mg/ml) and 100 mM (19.4 mg/ml). d-(+)-catechin hydrate, purchased from Nacalai Tesque Inc. (Kyoto, Japan), was dissolved in the external solution at the final concentrations of 1 mM (290 μg/ml), 2.5 mM (726 μg/ml), 5 mM (1.45 mg/ml), 10 mM (2.90 mg/ml) and 25 mM (7.26 mg/ml). As we described previously [9–13], bright-field images were obtained from randomly chosen 0.1-mm^2^ fields of view (10 views from each condition). Using the differential-interference contrast (DIC) microscopy, we counted degranulated mast cells and calculated their ratio to all mast cells (Table 1). Degranulated mast cells were defined as cells surrounded by more than 8 granules outside the cell membrane as described previously [14]. Data were analyzed by Microsoft Excel (Microsoft Corporation, Redmond, Wash., USA) and reported as means ± SD. Statistical significance was assessed by two-way ANOVA. A value of *p* < 0.05 was considered significant.

**Table 1:** Summary of mast cell counts in external solutions containing caffeine or catechin

Substances	Degranulating mast cell counts/view	All mast cell counts/view	Degranulating mast cell ratio (%)
External solution (control)	26.0 ± 3.30	27.4 ± 4.01	95.4 ± 7.70
1 mM caffeine	84.3 ± 13.7	86.2 ± 14.7	97.9 ± 1.50
5 mM caffeine	59.8 ± 17	61.8 ± 17.7	96.6 ± 2.88
10 mM caffeine	30.9 ± 14.9	46.7 ± 9.26	64.7 ± 23.3
25 mM caffeine	10.8 ± 3.99	49.6 ± 9.19	21.8 ± 6.96
50 mM caffeine	5.10 ± 2.28	36.0 ± 15.0	14.3 ± 3.70
100 mM caffeine	4.90 ± 2.64	45.2 ± 13.5	11.1 ± 5.62
External solution (control)	42.5 ± 8.67	43.5 ± 8.78	97.7 ± 2.56
1 mM catechin	81.2 ± 12.7	87.0 ± 14.3	93.5 ± 3.23
2.5 mM catechin	35.0 ± 5.21	39.0 ± 6.32	90.0 ± 3.83
5 mM catechin	50.0 ± 7.16	60.0 ± 11.1	84.1 ± 6.47
10 mM catechin	33.5 ± 10.1	43.2 ± 12.4	77.4 ± 9.18
25 mM catechin	17.8 ± 6.70	49.7 ± 10.4	35.5 ± 10.7
External solution (control)	66.8 ± 19.1	68.5 ± 19.0	97.4 ± 1.80
10 mM caffeine	17.7 ± 13.8	35.7 ± 10.7	46.2 ± 26.1
10 mM caffeine + 1 mM catechin	16.6 ± 10.8	72.6 ± 22.3	22.4 ± 10.9
10 mM caffeine + 2.5 mM catechin	14.2 ± 4.66	58.3 ± 15.3	24.9 ± 8.38

Values are means ± SD

Mast cells incubated in the external solution alone or relatively lower concentrations of caffeine (1, 5 mM) showed many wrinkles on their cell surface and released secretory granules as a result of exocytosis Fig. 1Ab–d vs. a). However, in mast cells incubated in relatively higher concentrations of caffeine (10, 25, 50, 100 mM), such findings suggestive of exocytosis were partially or completely absent (Fig. 1Ae, f, g, h). Quantitatively, relatively lower concentrations of caffeine (1, 5 mM) did not affect the numbers of degranulating mast cells (Fig. 1B). However, 10 mM caffeine significantly decreased the number of degranulating mast cells (control, 95.4 ± 7.70% vs. 10 mM caffeine, 64.7 ± 23.3%; *n* = 10, *P* < 0.05), and concentrations equal to or higher than 25 mM more markedly reduced the numbers of degranulating cells (25 mM caffeine, 21.8 ± 6.96%; 50 mM caffeine, 14.3 ± 3.70%; 100 mM caffeine, 11.1 ± 5.62%; *n* = 10, *P* < 0.05; Fig. 1B). Similarly to the findings obtained from caffeine, relatively lower concentrations of catechin (1, 2.5 mM) did not affect the degranulation of mast cells (Fig. 2Ab, c, d) and the numbers of which were almost comparable to those incubated in the external solution alone (Fig. 2B). However, catechin with concentrations equal to or higher than 5 mM partially or entirely halted the process of exocytosis (Fig. 2Ae, f, g) and significantly suppressed the numbers of degranulating mast cells (control, 97.7 ± 2.56% vs. 5 mM catechin, 84.1 ± 6.47%; 10 mM catechin, 77.4 ± 9.18%; *n* = 10, *P* < 0.05; Fig. 2B). Twenty-five mM catechin alone showed a marked reduction of the number of degranulating mast cells (35.5 ± 10.7%, *n* = 10, *P* < 0.05).

**Fig. 1:**
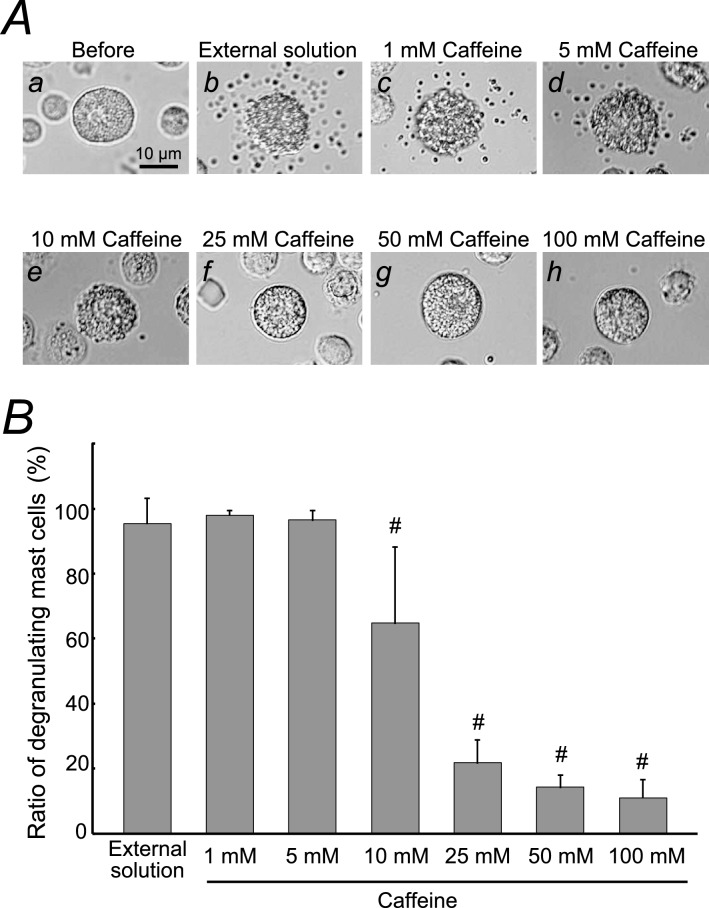
Effects of caffeine on mast cell degranulation. **A** Differential-interference contrast (DIC) microscopic images were taken before (a) and after exocytosis was externally induced by compound 48/80 in mast cells incubated in the external solutions containing no caffeine (b), 1 mM caffeine (c), 5 mM caffeine (d), 10 mM caffeine (e), 25 mM caffeine (f), 50 mM caffeine (g) and 100 mM caffeine (h). **B** After the mast cells were incubated in the external solutions containing no caffeine or different concentrations (1, 5, 10, 25, 50 and 100 mM) of caffeine, exocytosis was induced by compound 48/80. The numbers of degranulating mast cells were expressed as percentages of the total mast cell numbers in selected bright fields. ^**#**^*p* < 0.05 vs. incubation in the external solution alone. Values are means ± SD. Differences were analyzed by ANOVA followed by Dunnett’s *t* test

**Fig. 2:**
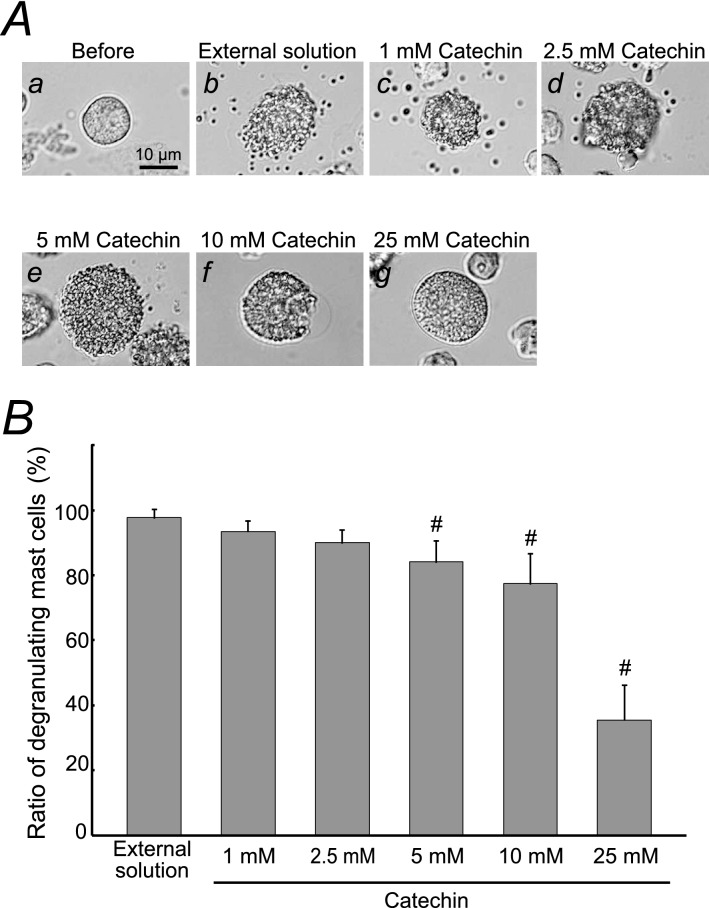
Effects of catechin on mast cell degranulation. **A** Differential-interference contrast (DIC) microscopic images were taken before (a) and after exocytosis was externally induced by compound 48/80 in mast cells incubated in the external solutions containing no catechin (b), 1 mM catechin (c), 2.5 mM catechin (d), 5 mM catechin (e), 10 mM catechin (f) and 25 mM catechin (g). **B** After the mast cells were incubated in the external solutions containing no caffeine or different concentrations (1, 2.5, 5, 10 and 25 mM) of catechin, exocytosis was induced by compound 48/80. The numbers of degranulating mast cells were expressed as percentages of the total mast cell numbers in selected bright fields. ^**#**^*p* < 0.05 vs. incubation in the external solution alone. Values are means ± SD. Differences were analyzed by ANOVA followed by Dunnett’s *t* test

Previous studies indirectly determined the mast cell-stabilizing properties of caffeine or catechin by measuring the amount of chemical mediators released from mast cells [5, 6, 15]. However, besides their exocytotic release of chemical mediators, including histamine, leukotrienes or β-hexosaminidase, mast cells generate various types of inflammatory cytokines or growth factors [16]. In this regard, to accurately define the ability of caffeine or catechin on the stabilization of mast cells, the release of all these substances have to be evaluated. Otherwise, the exocytotic process itself has to be monitored directly in mast cells. In the present study, we carefully observed the whole process of exocytosis under the microscope and actually counted the numbers of degranulating mast cells. Thus, we provided direct evidence for the first time that both caffeine and catechin dose-dependently inhibited the process of exocytosis and thereby exerted mast cell-stabilizing properties.

From our results, despite the lack of statistical significance, relatively lower concentrations of catechin (1, 2.5 mM) tended to decrease the numbers of degranulating mast cells (Fig. 2B). In our recently study, low dose prazosin, an α_1_-adrenergic receptor blocker, potentiated the ability of adrenaline, the first choice medication for anaphylaxis, to stabilize mast cells [13]. Therefore, expecting the similar additive therapeutic efficacy by low dose substances, we examined the effects of 1 or 2.5 mM catechin on the caffeine-induced inhibition of exocytosis (Fig. 3). Consistent with our results shown in Fig. 1B, 10 mM caffeine significantly but not markedly reduced the number of degranulating mast cells (control, 97.4 ± 1.80% vs. 10 mM caffeine, 46.2 ± 26.1%; *n* = 10, *P* < 0.05; Fig. 3B). However, surprisingly enough, in the presence of 1 or 2.5 mM catechin, the exocytotic process of mast cells was almost completely halted (Fig. 3Ac, d vs. b). Regarding the numbers of degranulating mast cells, they were more markedly decreased than those with 10 mM caffeine alone (10 mM caffeine + 1 mM catechin, 22.4 ± 10.9%, *n* = 34, *P* < 0.05; 10 mM caffeine + 2.5 mM catechin, 24.9 ± 8.38%, *n* = 10, *P* < 0.05; Fig. 3B), showing that the inhibitory effect of caffeine on exocytosis was augmented. These results suggested that lower concentrations of catechin can synergistically potentiate the mast cell-stabilizing property of caffeine.

**Fig. 3:**
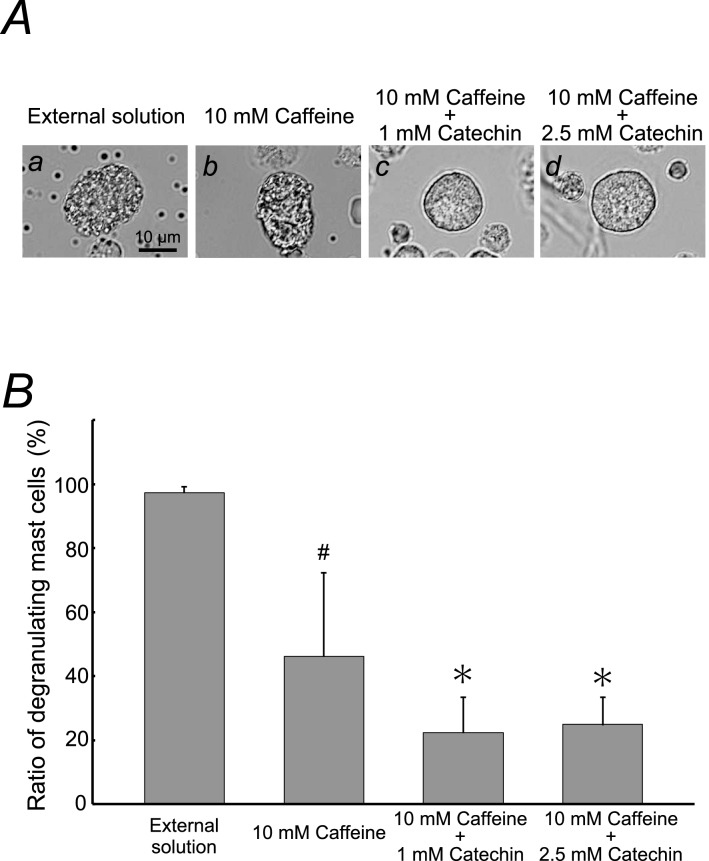
Effects of low concentrations of catechin on caffeine-induced inhibition of mast cell degranulation. **A** Differential-interference contrast (DIC) microscopic images were taken after exocytosis was externally induced by compound 48/80 in mast cells incubated in the external solutions containing no substances (a), 10 mM caffeine alone (b), 10 mM caffeine in the presence of 1 mM catechin (c) and 10 mM caffeine in the presence of 2.5 mM catechin (d)*. B* After exocytosis was induced in mast cells incubated in the external solutions containing no substances, 10 mM caffeine alone, 10 mM caffeine in the presence of 1 or 2.5 mM catechin, the numbers of degranulating mast cells were expressed as percentages of the total mast cell numbers in selected bright fields. ^**#**^*p* < 0.05 vs. incubation in the external solution alone. *p < 0.05 vs. incubation in the external solution containing 10 mM caffeine alone. Values are means ± SD. Differences were analyzed by ANOVA followed by Tukey’s test

In mast cells, as we demonstrated in patch-clamp studies [10], an increase in the intracellular Ca^2+^ concentration ( [Ca^2+^]_i_) is the primary trigger of exocytosis [17]. According to several in vitro studies, caffeine and catechin suppress the elevation of [Ca^2+^]_i_ either directly or through the activation of adenylate cyclase and the subsequent increase in the intracellular cyclic adenosine monophosphate (cAMP) [5, 6]. Recently, Nishikawa et al. additionally revealed the involvement of reactive oxygen species (ROS) in the effect of catechin on mast cell degranulation [15]. In their study, catechin exerted dual effects depending on the levels of intracellular ROS. When intracellular ROS level was low, catechin paradoxically suppressed the degranulation of mast cells. In the present study, 1 or 2.5 mM catechin alone was not enough to decrease the numbers of degranulating mast cells (Fig. 2B). However, in the presence of 10 mM caffeine, a potent scavenger of ROS [18], these concentrations of catechin remarkably reduced the numbers of degranulating mast cells (Fig. 3B). From these findings, caffeine-induced decrease in the intracellular ROS level was thought to elicit the ability of low concentrations of catechin to stabilize mast cells, which in turn synergistically potentiated the mast cell-stabilizing property of caffeine itself.

In our series of patch-clamp studies, by detecting the changes in whole-cell membrane capacitance (Cm) in mast cells, we provided electrophysiological evidence that anti-allergic drugs, anti-microbial drugs and corticosteroids inhibit the process of exocytosis, and thus exert mast cell-stabilizing properties [9–13]. Using the same approach in the future, we could more elaborately determine the mast cell-stabilizing property of caffeine or catechin.

In summary, this study provided direct evidence for the first time that caffeine and catechin dose-dependently inhibit the process of exocytosis. At relatively lower concentrations, caffeine or catechin alone did not stabilize mast cells. However, low concentrations of catechin synergistically potentiated the mast cell-stabilizing property of caffeine.

## Data Availability

The data used to support the findings of this study are available from the corresponding author upon request.
